# Temporal Unmixing of Dynamic Fluorescent Images by Blind Source Separation Method with a Convex Framework

**DOI:** 10.1155/2015/713424

**Published:** 2015-05-24

**Authors:** Duofang Chen, Jimin Liang, Kui Guo

**Affiliations:** School of Life Science and Technology, Xidian University, Xi'an, Shaanxi 710071, China

## Abstract

By recording a time series of tomographic images, dynamic fluorescence molecular tomography (FMT) allows exploring perfusion, biodistribution, and pharmacokinetics of labeled substances in vivo. Usually, dynamic tomographic images are first reconstructed frame by frame, and then unmixing based on principle component analysis (PCA) or independent component analysis (ICA) is performed to detect and visualize functional structures with different kinetic patterns. PCA and ICA assume sources are statistically uncorrelated or independent and don't perform well when correlated sources are present. In this paper, we deduce the relationship between the measured imaging data and the kinetic patterns and present a temporal unmixing approach, which is based on nonnegative blind source separation (BSS) method with a convex analysis framework to separate the measured data. The presented method requires no assumption on source independence or zero correlations. Several numerical simulations and phantom experiments are conducted to investigate the performance of the proposed temporal unmixing method. The results indicate that it is feasible to unmix the measured data before the tomographic reconstruction and the BSS based method provides better unmixing quality compared with PCA and ICA.

## 1. Introduction

Allowing noninvasive, quantitative, and three-dimensional (3D) imaging of fluorescence probes associated with molecular and cellular functions [1–3], fluorescence molecular tomography (FMT) has been applied to drug development [4] and preclinical oncological research [2, 5–8]. By recording a sequence of tomographic images at intervals of a few minutes, dynamic FMT allows capturing the metabolic processes of fluorescent biomarkers [9–13], which is helpful in better understanding the complete dynamic course or pharmacokinetics including inflow, uptake, and washout of fluorescent biomarkers (or drugs). Dynamic FMT can provide spatially resolved kinetics of an optical contrast agent and offers an attractive approach in studying drug delivery, tumor detection, and treatment monitoring [14].

However, the widespread adoption of dynamic FMT imaging has been inhibited by its inability to clearly resolve and identify metabolic processes of optical contrast agent throughout whole body of small animal in vivo [15, 16]. In recent years, several research groups have proposed solutions to the problem. The solutions assume that the fluorescent tomography images are a linear combination of different fluorescent sources images and mainly use blind source separation (BSS) based methods. In [17], Hillman and Moore demonstrate that PCA is capable of extracting anatomical information of various internal organs using 2D dynamic fluorescence reflectance imaging. In [15], PCA is used to detect and visualize changes in kinetic behaviors between different regions from 3D dynamic FMT images. In [16], Liu et al. propose an ICA-based method to unmix dynamic FMT images and the detecting capability of ICA is improved compared with PCA. In [18], pharmacokinetic-rate images of optical fluorophores are directly reconstructed from NIR measurements, which implies that it is possible to reconstruct the tomographic images from the unmixed measured images rather than unmixing the reconstructed tomographic images. In [19], PCA is directly applied to the fluorescence projection sequence, which leads to a reduced computation cost while a similar resolving capability was compared with the method depicted in [15]. However, PCA and ICA utilize the statistical property that the sources are mutually uncorrelated or independent, supposing the sources do satisfy that property. They may fail when the sources do not satisfy the above property. In this paper, we present a nonnegative BSS based method, named as convex analysis of mixtures of nonnegative sources (CAMNS), to separate the observed fluorescent image sequence. CAMNS requires no assumption on source independence or zero correlations and obtains positive or zero results [20].

This paper is organized as follows. First, the linear relationship between the measurements and the kinetic course of fluorophore is established for dynamic FMT based on the finite element method (FEM); second, the nonnegative blind source separation method CAMNS is introduced to unmix the observed images and extract the fluorescent sources according to the linear relationship; and finally numerical simulation and phantom experiments are performed to evaluate the efficacy of the proposed method.

## 2. Materials and Methods

### 2.1. Linear Relationship Establishment for Dynamic FMT

For continuous wave FMT, the photon propagation at the excitation and emission wavelengths can be modeled by the following coupled diffusion equations (DE) [21–23]:

(1) ∇·Dxr∇Φxr−μaxrΦxr=−qr,∇·Dmr∇Φmr−μamrΦmr=−ημafrΦxrr∈Ω,

where subscripts *x* and *m* denote the excitation and emission wavelengths, respectively. Φ_*x*,*m*_(*r*) represents the spatially varying photon density in the medium. *D*_*x*,*m*_(*r*) is the diffusion coefficient and *μ*_*ax*,*m*_(*r*) stands for the absorption coefficient. *ημ*_*af*_(*r*) is the fluorescent yield and is denoted as **x** in the following part of this paper. The absorption coefficient *μ*_*af*_(*r*) is proportional to the fluorophore concentration by the formula *μ*_*af*_(*r*) = ln10*εC*(*r*), where *ε* is the molar extinction coefficient and *C*(*r*) is the concentration of the fluorophore [24]. Using the Robin-type boundary conditions [23, 25] and the finite element method, we have the final weighted matrix:

(2) $$\begin{array}{c} \varphi =Ax. \end{array}$$

Equation (2) shows that a linear relationship exists between the measured photon flux density **φ** and the unknown fluorescent yield **x**. Readers are referred to [21, 26, 27] for more detailed descriptions.

In dynamic FMT, a series of boundary measurements are obtained to recover and analyse the time-varying fluorophore concentration. Usually, the time-varying fluorophore concentration in a special organ or tissue is complicated and related to the pharmacokinetics of a certain dye or drug in that organ or tissue [14, 17], and the pharmacokinetics parameters of fluorescent dye or drug in different organs or tissues are different. This is the assumption for detection and visualization of functional structures based on the reconstruction results of dynamic measurements [18]. In this paper, we define the fluorescent dye or drug in one organ or tissue as one light source. Assuming that there only exists one light source in the medium and the dynamic measurements are collected at time instances, *t*_1_, *t*_2_,…, *t*_*K*_, then the *K* measurements can be depicted in the following form:

(3) φt1,φt2,…,φtK=Axt1,xt2,…,xtK=AX,

where **x**(*t*_*k*_) is the fluorescent yield at *t*_*k*_, **φ**(*t*_*k*_) is a *N* × 1 vector, and *N* is the number of pixels in each acquired image.

When we have to study the fluorophore distribution in more than one tissue, say *P* tissues, then there exist *P* light sources. Transposing the measured *N* × *K* data matrix to *K* rows and *N* columns matrix and taking the background and noise into consideration, we can get the relationship between the dynamic measurements and the fluorophore distribution as

(4) $$\begin{array}{c} \Psi =\overset{P}{\underset{p=1}{\sum }}A_{p}X_{p}+B+\varepsilon , \end{array}$$

where Ψ is the boundary measurements matrix of *K* rows and *N* columns and the *k*th row represents the measurement at time instance *t*_*k*_. **A**_*p*_ and **X**_*p*_ are the system matrix and the fluorescent yield series corresponding to the *p*th light source, respectively. **B** indicates the background signal and **ε** the noise. The background signal can be easily removed through subtracting the measured images by a preinjection image. So in the subsequent sections, we suppose that the measured data are free of background signal unless otherwise specified. The noise **ε** can also be ignored considering that the EMCCD camera is cooled to −80 degrees during data measurement, and the dark current and read noise is about several electrons [28]. From (4) it is obvious that the unmixing can be operated directly on the observed images.

### 2.2. Temporal Unmixing of Dynamic Images by CAMNS

In dynamic fluorescence molecular tomography study, a sequence of photon density images is acquired at intervals of minutes over time. Blind source separation methods, such as PCA and ICA, provide capability of recovering hidden sources (fluorescent biomarkers or functional structures) from these acquired images. However, PCA and ICA perform well only when the sources are mutually uncorrelated or independent. Considering that the recovered sources are nonnegative by nature, we adopt a nonnegative blind source separation framework, known as CAMNS to study dynamic images. CAMNS adopts a special deterministic assumption called local dominance which means that the true source signals serve as the extreme points of some observation-constructed polyhedral set [20].

According to the framework proposed in [20], the measurements Ψ can be considered as a polyhedral set whose extreme points are **A**_1_ **X**_1_, **A**_2_ **X**_2_,…, and **A**_*P*_ **X**_*P*_. And **A**_*p*_ **X**_*p*_ is the surface photon flux density from the *p*th inner light source. By finding all the extreme points of the polyhedral set Ψ, the true sources can be perfectly identified. The extreme points can be determined by solving the following linear programs:

(5) p∗=minβ ⁡rTHβ+dsubject  to  Hβ+d≥0q∗=maxβ ⁡rTHβ+dsubject  to  Hβ+d≥0,

where **r** is a random uniform vector and matrix **H** and vector **d** are both obtained from the observations and are defined as

(6) d=1NΨ1N×1H=u1,u2,…,uP−1,

where 1_*N*×1_ is an *N* × 1 all one vector. **u**_1_, **u**_2_,…, **u**_*P*−1_ are the *P* − 1 eigenvectors associated with the *P* − 1 principle eigenvalues of the following correlation matrix:

(7) $$\begin{array}{c} C_{\Psi }=\frac{1}{N}\left(\;\Psi -D\;\right){\left(\;\Psi -D\;\right)}^{T}, \end{array}$$

where **D** = [**d**, **d**,…, **d**] is a *K* × *N* matrix.

To get *P* source vectors, (5) should be solved for *P* times and *P* extreme points will be obtained. The theoretical proof and solving procedure of the linear programming problems are depicted in detail in [20].

### 2.3. Determination of Source Number

To find the *P* extreme points of the polyhedral set Ψ, the number of sources should be known as a prior. Eigenvalue decomposition is a commonly used method to determine the target number in phased-array radar signal processing [29]. To get the number of sources, the correlation matrix of the measured data is first calculated according to (7) and then the eigendecomposition is performed on the correlation matrix:

(8) $$\begin{array}{c} C_{\Psi }U=U\Sigma , \end{array}$$

where matrix **U** is composed of the eigenvectors and contains dynamic information of fluorescence sources and diagonal matrix Σ gives the ordered eigenvalues. We can determine the number of sources hided in the measured data by looking at the first nonnegligible eigenvalues.

### 2.4. Evaluation of the Unmixing Approaches

To quantify the deviation of the unmixed image from the true image, the rooted mean squared error (RMSE) is defined as follows:

(9) $$\begin{array}{c} \delta =\sqrt{\overset{P}{\underset{p=1}{\sum }}\;\overset{N}{\underset{n=1}{\sum }}{\left({\overset{-}{y}}_{pn}-{\overset{-}{s}}_{pn}\right)}^{2}}, \end{array}$$

where ${\overset{-}{y}}_{pn}$ and ${\overset{-}{s}}_{pn}$ are the *n*th element of the normalized separated image ${\overset{-}{y}}_{p}$ and the normalized true image ${\overset{-}{s}}_{p}$, respectively for the *p*th source, and the normalized images are obtained by ${\overset{-}{y}}_{p}=y_{p}/max(y_{p})$ and ${\overset{-}{s}}_{p}=s_{p}/max(s_{p})$. Due to the intrinsic feature of the blind source separation approach, the unmixed results are not unique, so the mean squared error is calculated based on the normalized images. In addition, all the unmixed results shown in the following results and discussion section are in the normalized way.

As described above, the proposed temporal unmixing method for dynamic images mainly includes the following four steps.

1. Organize the observed dynamic data in the form presented by (4) based on the finite element method.
2. Remove background signal from the measured data.
3. Perform eigenvalue decomposition to the correlation matrix of the measured data with no background and determine the source number according to the number of nonnegligible eigenvalues.
4. Solve the two liner programs of (5) and the hidden sources images can be extracted.

### 2.5. Experimental Setup and Materials

The performance of the proposed approach was investigated by both numerical simulation and phantom experiments.

**Figure 1:**
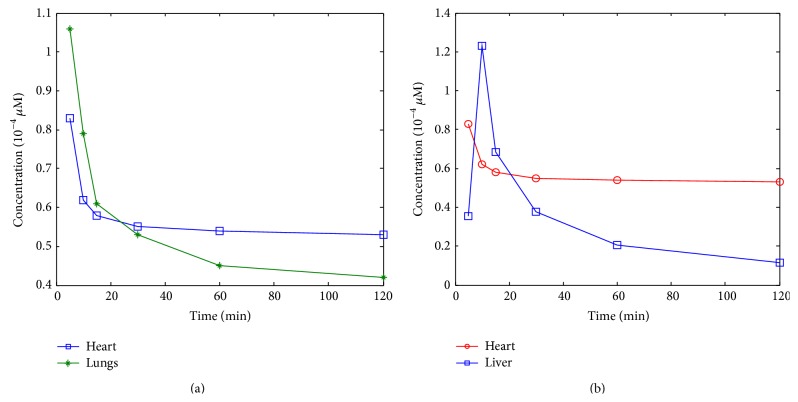
ICG concentration time courses used in (a) Simulation 1 and (b) Simulation 2. The markers depict actual concentration values at corresponding time points according to [30].

Three numerical simulations were conducted to evaluate the efficacy of the method under different conditions. In Simulation 1, the biodistribution of ICG in the heart and the lungs of a digital mouse was simulated according to [30]. The optical parameters are listed in Table 1. First, we set a series of fluorescent yields (concentrations of ICG) to the heart and the lungs at six time points (5 min, 10 min, 15 min, 30 min, 60 min, and 120 min), and the photon density on the mouse surface was simulated using the FEM approach. Second, the ICG distribution in the heart and the lungs was reconstructed. Finally, the mixed tomographic images were separated by PCA, ICA, and CAMNS, respectively. The ICG concentrations versus time, called time courses, are shown in Figure 1(a). The correlation coefficient between the two sources defined as $r=\sum_{n=1}^{N}\left(s_{1n}-{\overset{-}{s}}_{1}\right)\left(s_{2n}-{\overset{-}{s}}_{2}\right)/\sqrt{\sum_{n=1}^{N}{\left(s_{1n}-{\overset{-}{s}}_{1}\right)}^{2}{\left(s_{2n}-{\overset{-}{s}}_{2}\right)}^{2}}$ is 0.64, which means the sources are correlated. In Simulation 2, the biodistribution of ICG in the heart and the liver of a digital mouse were used as the sources. The time courses of the two sources are shown in Figure 1(b). The correlation coefficient between the two sources is 0.01, which means that they are uncorrelated. The optical parameters are the same as those in Simulation 2. In Simulation 3, in order to test the robustness of CAMNS, we added Gaussian noise of zero mean and varying variance to the observations used in Simulation 2. The varying variance of the Gaussian noise is 1, 10^−1^, 10^−2^, 10^−3^, 10^−4^, 10^−5^, and 10^−6^, respectively, while the maximum amplitude of the clean images is 1. For every variance, 100 Monte Carlo (MC) tests were run and the RMSEs defined by (9) were calculated.

**Figure 2:**
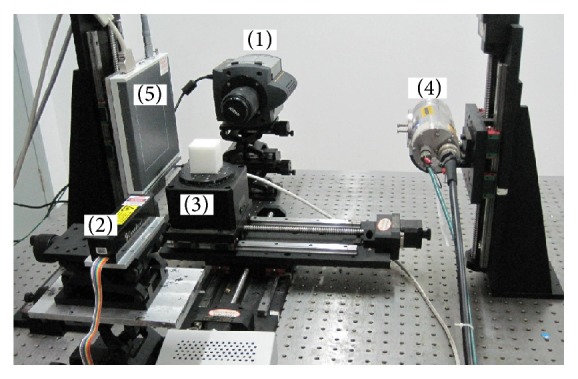
The FMT/MicroCT dual modality imaging system: (1) EMCCD; (2) diode laser; (3) rotational stage; (4) X-ray tube; and (5) X-ray detector.

In the phantom experiments, the prototype FMT/MicroCT dual modality imaging system, as shown in Figure 2, was used to collect the fluorescent data. The imaging system contains mainly three parts. One is the FMT subsystem which consists of a diode laser (CL-671-050, CrystaLaser, Nevada, USA) and a Nikkor 40 mm f2.8 lens (Nikon, Melville, New York, USA) coupled EMCCD camera (Andor iXon3, Belfast, UK). Another is the rotational stage where the phantom is placed. And the last one is the MicroCT subsystem which consists of an X-ray tube (OXFORD INSTRUMENTS series 5000 Apogee X-ray tube, X-ray technology Inc., CA) with a focal spot size of 35 *μ*m and a high-resolution flat panel X-ray detector (HAMAMATSU C7921CA-02, Hamamatsu city, Japan) incorporating a 1032 × 1012 pixel photodiode array with a 50 *μ*m pixel pitch.

Three phantom experiments were conducted to evaluate the performance of the proposed method and two phantoms were utilized. The phantoms are made from polyoxymethylene and the optical parameters at the excitation and emission wavelengths are illustrated in Table 2 [26]. Phantom number 1 is 25 mm × 30 mm × 30 mm and five small holes with 2 mm radius are drilled and the distance between the two adjacent holes is 5 mm; phantom number 2 is cubic with side length of 25 mm and four small holes of 2 mm radius are drilled along the diagonal line and the distance between the two adjacent holes is about 7 mm.

**Figure 3:**
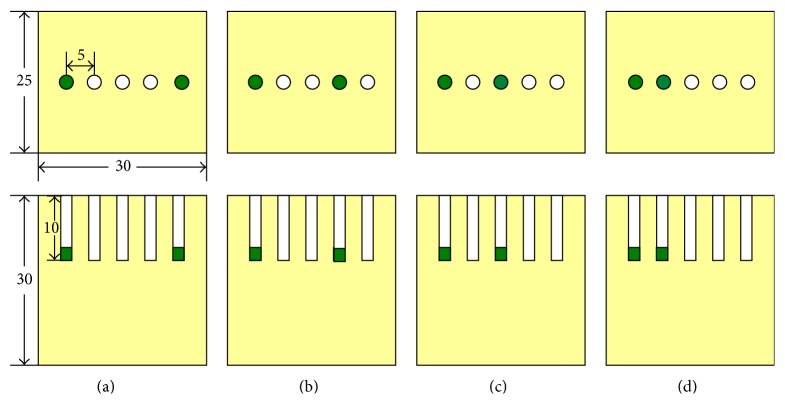
Top view and front view of source 1 and source 2 for phantom Experiment 1. From left to right: (a) case 1: 2 sources were 20 mm apart, (b) case 2: 2 sources were 15 mm apart, (c) case 3: 2 sources were 10 mm apart, and (d) case 4: 2 sources were 5 mm apart.

**Figure 4:**
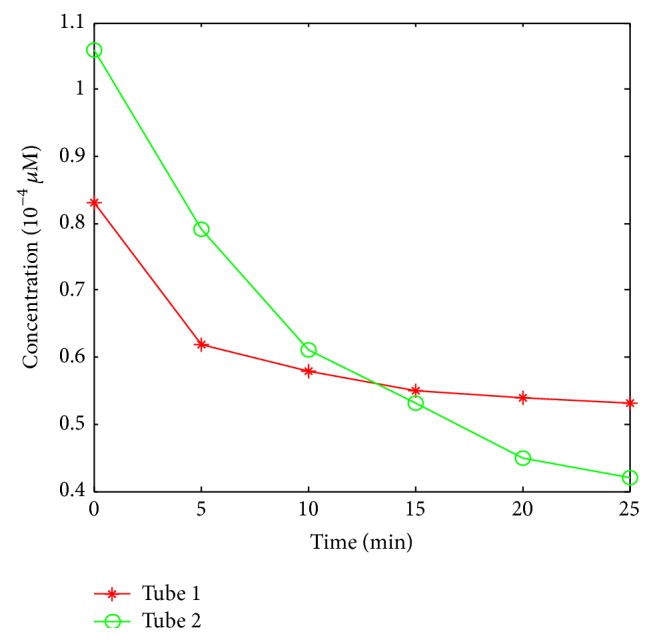
Time courses of the Cy5.5 dye in 2 tubes. Different colors correspond to concentrations in different tubes (red: tube 1 and green: tube 2).

**Figure 5:**
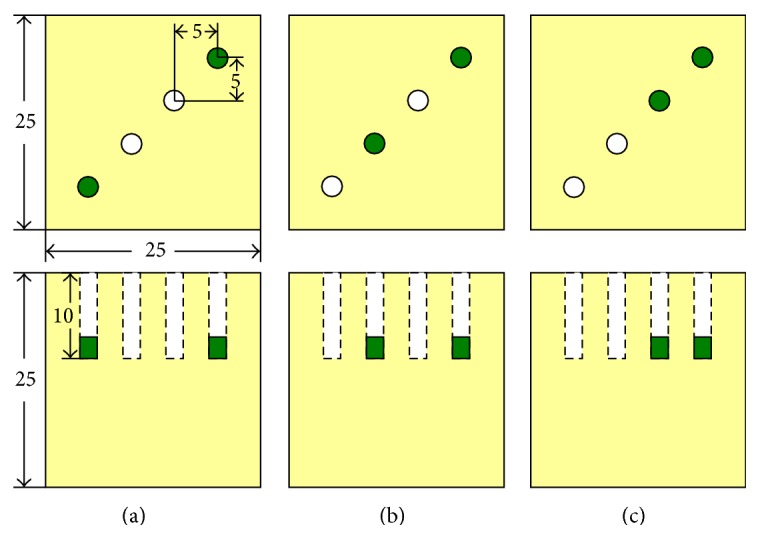
Top view and front view of 2 source locations in phantom Experiment 2, (a) 15 mm apart in depth, (b) 10 mm apart in depth, and (c) 5 mm apart in depth.

**Figure 6:**
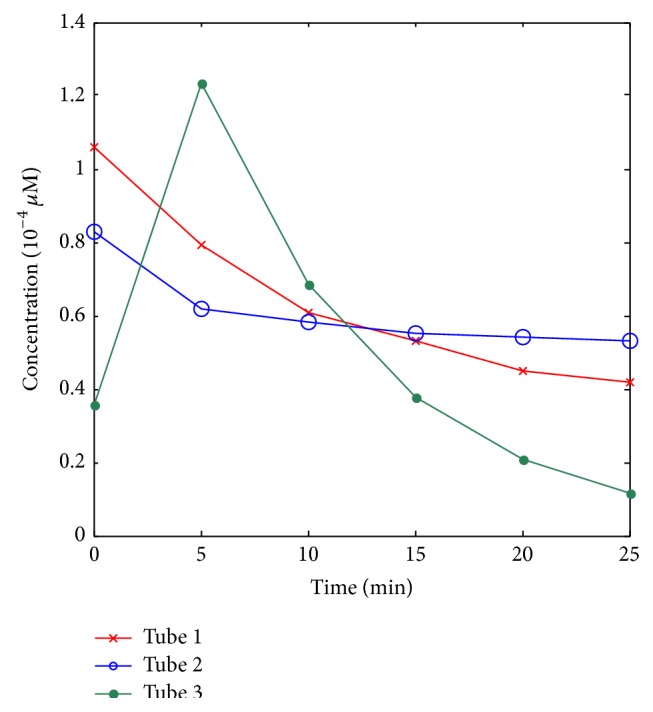
Time courses of Cy5.5 in three tubes.

In phantom Experiment 1, two of the five holes in phantom number 1 were used to emplace capillary tubes filled with Cy5.5 solution of different concentrations. The top view and the front view of the phantom are illustrated in Figure 3. Four cases were investigated. In case 1, the two tubes were placed 20 mm apart from each other; in case 2, the two tubes were placed 15 mm apart from each other; in case 3, the two tubes were placed 10 mm apart from each other; and in case 4, the two tubes were placed 5 mm apart from each other. The hole displayed in green color means that this hole has Cy5.5 solution. The time courses of the Cy5.5 dye at six time points are shown in Figure 4. In phantom Experiment 2, as shown in Figure 5, two of the four holes in phantom number 2 were used to emplace capillary tubes filled with Cy5.5 solution of different concentrations. Three cases were investigated in this experiment. In case 1, both the lateral distance and the vertical depth between the 2 sources were 15 mm; in case 2, both the lateral distance and the vertical depth between the 2 sources were 10 mm; in case 3, both the lateral distance and the vertical depth between the two sources were 5 mm. The top and front views of the phantom are illustrated in Figure 8 and the green color means that this hole has fluorescent dye. The dye concentrations are the same as those in Experiment 1 which are shown in Figure 4. In phantom Experiment 3, the capability of the method to separate more than two sources, for example, three sources, was studied. To simulate three sources, three capillary tubes of Cy5.5 solutions were put into three holes of phantom number 1. And the tubes were 10 mm away from the adjacent ones. The time courses of the Cy5.5 solution in the three capillary tubes are plotted in Figure 6.

During each phantom experiment, the phantom was placed on the rotational stage, which was controlled by a computer. The excitation illumination was provided by a 671 nm CW diode laser with a power of 3 mW. A 35 nm band-pass filter centered at 720 nm was placed in front of the camera to allow light transmission at the emission wavelength. The EMCCD camera was cooled to −80°C when collecting the fluorescent signals frame by frame.

## 3. Results and Discussion

### 3.1. Simulation Study

In this section, three simulation results are presented. Simulation 1 is to separate two correlated sources, Simulation 2 is to extract two uncorrelated sources, and Simulation 3 is to unmix uncorrelated sources with Gaussian additive noise.

**Simulation 1 (correlated sources separation).**

**Figure 7:**
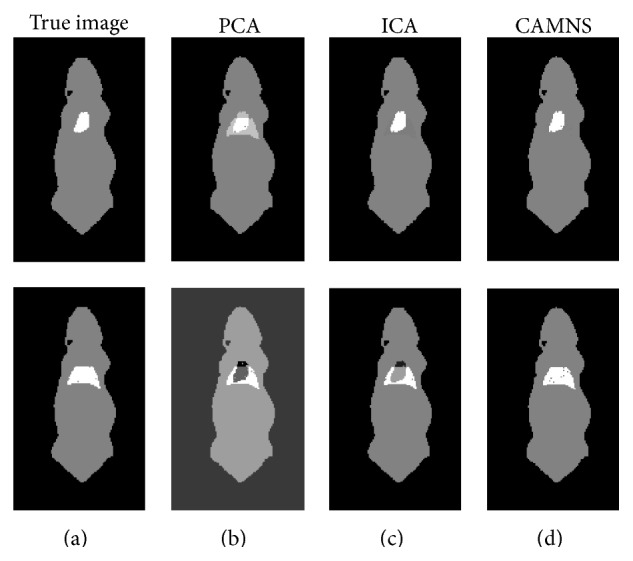
Temporal unmixing results for two organs (heart and lungs) in the mouse: (a) the true images and the unmixed results by (b) PCA, (c) ICA, and (d) CAMNS.

The ICG biodistribution in the heart and the lungs of a digital mouse at six time points was simulated. The sources are correlated and the correlation coefficient is 0.64. The ordered eigenvalues of the correlation matrix are 0.0000, 0.0000, 0.0000, 0.0001, and 0.0532, according to which the number of the sources in the mouse is set to be 2. The normalized separated results by PCA, ICA, and CAMNS are illustrated in Figure 7. The corresponding RMSEs for PCA, ICA, and CAMNS are 9.5736, 4.2500, and 0.2250, respectively. As PCA and ICA need the sources to be statistically uncorrelated or independent, they do not perform as well as CAMNS does.

**Simulation 2 (uncorrelated sources separation).**

**Figure 8:**
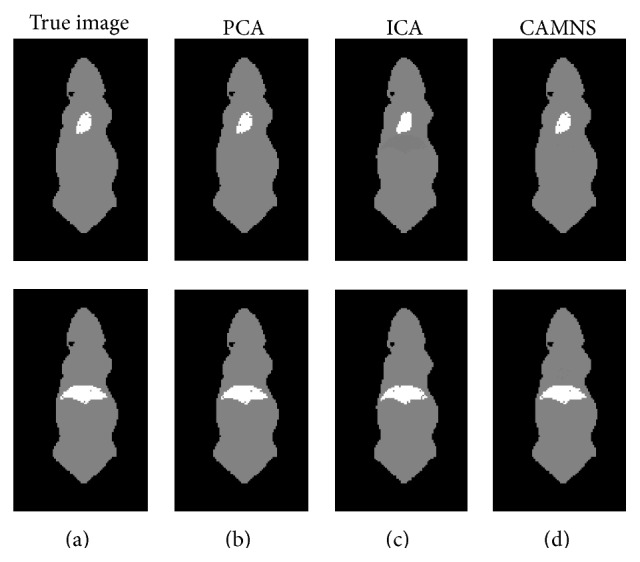
Temporal unmixing results for two organs (heart and liver) in the mouse: (a) the true images and the extracted images by (b) PCA, (c) ICA, and (d) CAMNS.

The ICG biodistribution in the heart and the liver of a digital mouse at six time points were simulated, where the heart and liver images were taken as the sources. The correlation coefficient between the two sources is 0.01, which means that they are uncorrelated. The ordered eigenvalues of the correlation matrix are 0.0000, 0.0000, 0.0000, 0.0000, 0.0047, and 0.0406, so the number of sources is set to be 2. The extracted sources obtained by PCA, ICA, and CAMNS are shown in Figure 8. The RMSEs for PCA, ICA, and CAMNS are 0.0001, 0.0002, and 0.0001, respectively. We can see that all these methods can work well when the sources are uncorrelated.

**Simulation 3 (uncorrelated sources separation under noisy environment).**

**Figure 9:**
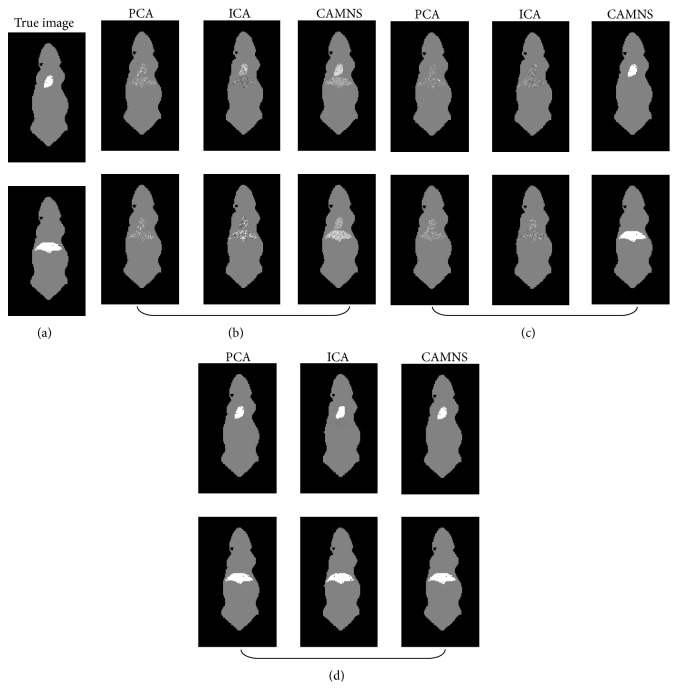
Uncorrelated sources separation under noisy condition: (a) the true images and the separated images by PCA, ICA, and CMANS when PSNR is (b) 0 dB, (c) 30 dB, and (d) 50 dB.

**Figure 10:**
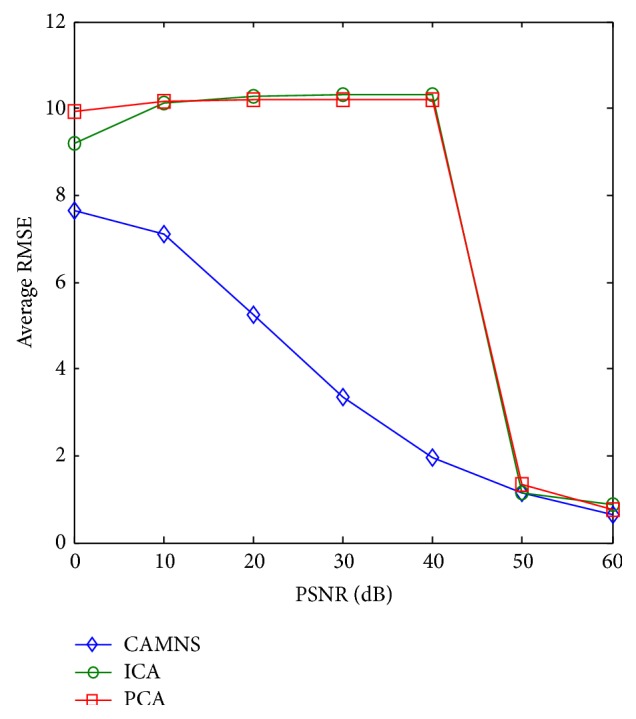
Average RMSEs by PCA, ICA, and CAMNS under noisy condition.

In this section, we investigate the robustness of PCA, ICA, and CAMNS under noisy environment. To exclude the effect of sources correlation, we use the same uncorrelated sources as those in Simulation 2. Gaussian noise with zero mean and varying variance is added. The variance of the Gaussian noise is 1, 10^−1^, 10^−2^, 10^−3^, 10^−4^, 10^−5^, and 10^−6^, while the maximum amplitude of the clean images is 1. For every variance, 100 Monte Carlo (MC) tests are run and the RMSEs defined by (9) are calculated. The separation results by PCA, ICA, and CAMNS of one MC test when PSNR is 0 dB, 30 dB, and 50 dB are shown in Figure 9. The average RMSEs over 100 MC tests for different PSNRs (defined here as 10log(*s* ^2^/*σ* ^2^) where *s* ^2^ is the peak power in the clean images and *σ* ^2^ is the variance of the Gaussian noise) are plotted in Figure 10. We can see that CAMNS extracts the sources successfully when PSNR reaches 30 dB, while PCA and ICA perform as well as CAMNS does when PSNR is no less than 50 dB. As shown in (7), the temporal frames are averaged to get the correlation matrix **C**_Ψ_. This averaging is equivalent to a mean filter, which can reduce the effect of noise. Benefitting from the averaging effect, CAMNS is more robust than PCA and ICA.

### 3.2. Phantom Experiments

In this section, three physical phantom experiments are presented to demonstrate the efficacy of the nonnegative blind source separation method for dynamic images analysis. Experiment 1 is to test the influence of source distance on the performance of the separation method. Experiment 2 is to further test the vertical spatial resolution of the method, so two sources with different lateral and vertical depth were used. And Experiment 3 is to evaluate the capability of the method to unmix more than two sources.

**Experiment 1 (two sources with different lateral distances).**

**Figure 11:**
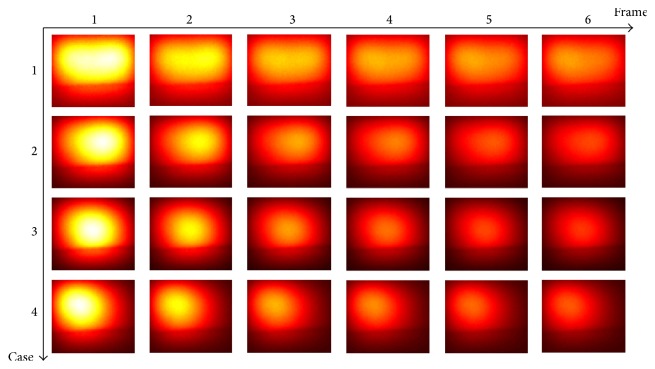
The mixed images captured by EMCCD camera. Six frames in a row are corresponding to six concentrations for one case.

The fluorescent images for six concentrations were captured from the front view and are shown in Figure 11, from which we can see that the two targets inside the phantom cannot be discriminated due to the intrinsic scattering and the limited spatial resolution. To get the background signal, we made a measurement when all the five holes are empty. Before temporal unmixing, all the observed images were subtracted by the background signal.

**Figure 12:**
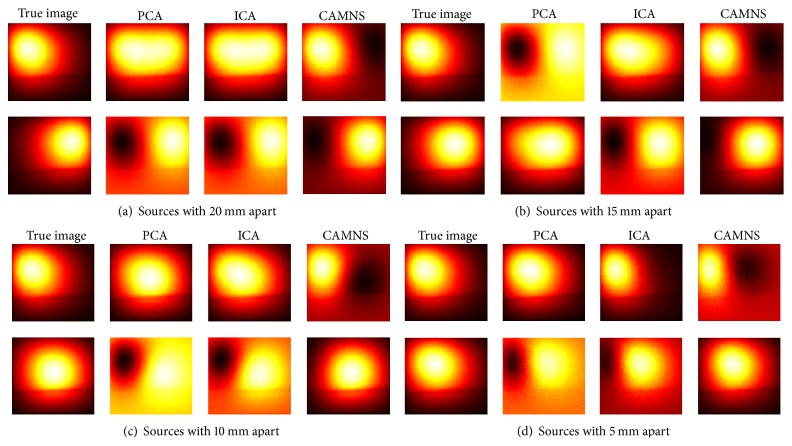
Temporal unmixing results for 2 sources of different distances. (a) Case 1: 20 mm apart, (b) case 2: 15 mm apart, (c) case 3: 10 mm apart, and (d) case 4: 5 mm apart.

The eigenvalues of the correlation matrix are listed in Table 3, from which the number of the fluorescent sources can be determined based on the number of nonnegligible values. According to Table 3, the number of fluorescent sources is set to be 2 for all the four cases. The true images and the temporal unmixed results by PCA, ICA, and CAMNS are shown in Figures 12(a)–12(d), which are corresponding to cases 1–4, respectively. Images in the first row are the results for source 1 and those in the second row for source 2. The true images are obtained by putting only one inclusion in one of the two holes. All the images are normalized for comparison. The performance of the temporal unmixing methods, evaluated by the rooted mean squared error, is given in Table 4.

It is shown that, for two sources of different lateral distances, the source number can be determined from the number of nonnegligible eigenvalues; see Table 3. From Figure 12 and Table 4, we can see that the unmixed results obtained by CAMNS are closer to the true ones and the RMSEs are smaller compared to the results obtained by PCA and ICA. The results by PCA and ICA are somewhat similar to each other in Figures 12(a)–12(d). We also find that the errors by PCA and ICA are not affected by the lateral distance, while the error by CAMNS decreases with the lateral distance. The reason may be that the theoretical basis of these methods is different from one another. For example, PCA is to find the orthogonal components and ICA is to find the independent components. And the orthogonality and the independence between the sources have no relation with the lateral distance. CAMNS is to find the extreme points of the observation-constructed polyhedral set. As the distance increases, the interference between the sources decreased and the number of pure pixel in the measured data increases. Hence the extracted extreme points maybe more accurate. The concept of pure pixel is referred to [31].

**Experiment 2 (two sources with different lateral distances and different vertical depths).**

**Figure 13:**
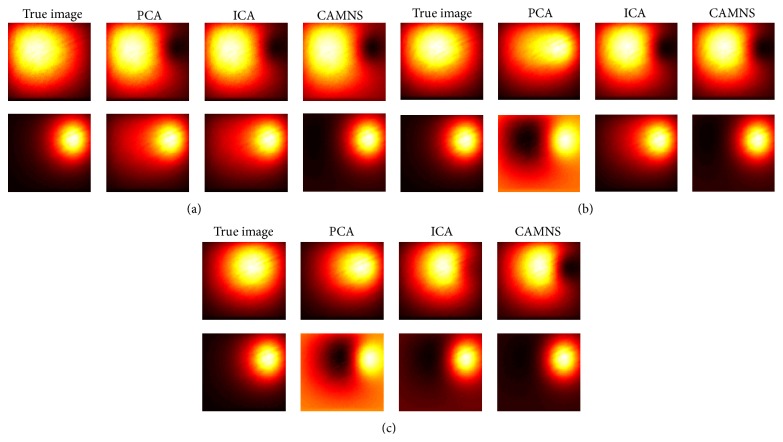
Temporal unmixing results for 2 sources of different lateral distances and vertical depths. (a) Case 1: 2 sources were 15 mm apart in depth, (b) case 2: 2 sources were 10 mm apart in depth, and (c) case 3: 2 sources were 5 mm apart in depth.

To further detect the efficacy of the proposed temporal unmixing method, sources of different lateral distances and different vertical depths were used. For two sources of different lateral and vertical distances, the source number can also be determined from the number of nonnegligible eigenvalues, which are not listed in this paper. As shown in Figure 13 and Table 5, the ICA and CAMNS have better performance than the PCA does, and the error by CAMNS is smaller than those by ICA. In Table 5, the mean squared errors by ICA and PCA in the first row are large because there are negative values in the unmixed images. While the CAMNS contain only nonnegative results, the error is much smaller. As the lateral distance and the vertical depth between the two sources increase, the performance of CAMNS becomes better, while PCA and ICA do not. The reason remains the same as that in phantom Experiment 1.

**Experiment 3 (three sources).**

**Figure 14:**
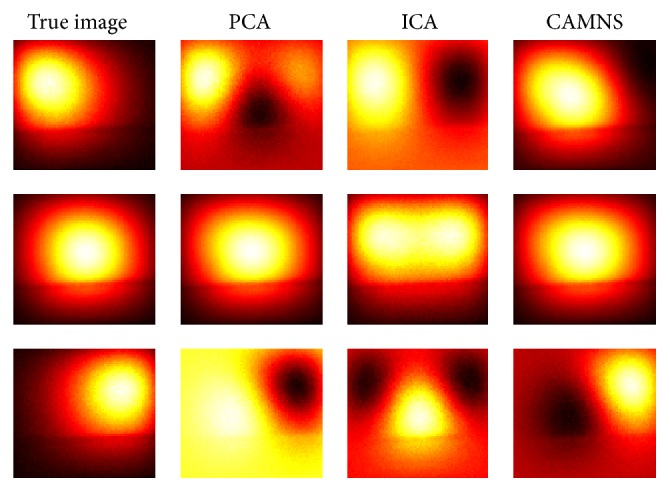
True images and unmixed images by PCA, ICA, and CAMNS for 3 sources. The first, the second, and the third row are corresponding to sources 1, 2, and 3, respectively.

From Experiments 1 and 2, we can see that the temporal unmixing method based on CAMNS performs better than PCA and ICA do for two sources. In this experiment, we investigate the capability of the method to separate three sources. The true images were captured by the EMCCD camera after putting one inclusion in one hole of the phantom.  Figure 14 shows the true images and the unmixed images obtained by PCA, ICA, and CAMNS. And the corresponding mean squared error for PCA, ICA, and CAMNS is 32.0566, 41.2831, and 19.6388, respectively. Again, the CAMNS gets the best results although the third source is a little different from the true one. The unmixed images got by PCA and ICA are deformed a lot compared with the true ones, which may be caused by the fact that there exist negative values in these images. From the results it may be concluded that the temporal unmixing based on CAMNS is more appropriate for situations where more than two targets exist. Similar to the assumption in phased-array signal processing, the maximum number of sources the method can discriminate properly should not be larger than the frames of the recorded images [29].

## 4. Conclusion

Dynamic FMT allows exploring perfusion, biodistribution, and pharmacokinetics of labeled substances in vivo by recording a time series of tomographic images. Usually, the image data sequence is measured first and then the dynamic tomographic images are reconstructed frame by frame and finally the unmixing operation based on PCA or ICA is performed to detect and visualize functional structures with different kinetic patterns. PCA and ICA utilize the statistical property that the sources are mutually uncorrelated or independent. When the sources satisfy these properties, PCA and ICA can get excellent results.

This paper focuses on the nonnegative temporal unmixing of the measured data in dynamic fluorescent molecular imaging. We deduce the relationship between the measured data sequence and the kinetic patterns using the finite element method and the relationship can be described in a linear form. Based on this liner relationship, the temporal unmixing can be performed on the measured data directly before the tomographic image reconstruction. The nonnegative BSS based method, named CAMNS, is presented to temporally unmix the measured data. CAMNS does not need sources to be uncorrelated or independent. It assumes that the sources are the extreme points of the polyhedral set constructed by the measurement and tries to find the extreme points. To evaluate the efficacy of the proposed method, three numerical simulations and three phantom experiments are conducted. In numerical simulation, PCA, ICA, and CAMNS are used to separate both correlated sources and uncorrelated sources. The results show that they all perform well to separate the uncorrelated sources while only CAMNS extracts the correlated sources successfully. We also study the performance of CAMNS under noisy condition. The results show the following. (a) PCA, ICA, and CAMNS fail to separate the sources when PSNR is less than 30 dB; (b) CAMNS can extract the sources correctly when PSNR reaches 30 dB; and (c) all the methods separate the sources successfully and the RMSEs are similar when PSNR is larger than 50 dB. In phantom experiments, PCA, ICA, and CAMNS are adopted to separate two sources of different distances. The case when three sources are present is also studied. The results show that CAMNS gets more accurate results compared with PCA and ICA. Although CAMNS performs better than PCA and ICA do, it needs to know the number of sources. We determine the number of sources by looking at the first nonnegligible eigenvalues of the measurement correlation matrix. PCA and ICA are recommended when the sources are uncorrelated and the noise is low. CAMNS is a better choice for dynamic FMT because this imaging modality is to explore the metabolic processes of fluorescent biomarkers inside small animals where the sources are usually correlated. In the future, we will pay attention to in vivo application of the algorithm.

## Figures and Tables

**Table 1:** Optical parameters of the digital mouse.

Organ	780 nm		830 nm	
	*μ*_*a*_ (mm^−1^)	*μ*_*s*_′ (mm^−1^)	*μ*_*a*_ (mm^−1^)	*μ*_*s*_′ (mm^−1^)
Muscle	0.038	0.280	0.028	0.235
Heart	0.027	0.776	0.021	0.710
Lungs	0.083	2.006	0.060	1.941
Liver	0.160	0.578	0.124	0.542
Kidney	0.030	1.791	0.023	1.631
Stomach	0.0053	1.240	0.0043	1.167

**Table 2:** Optical parameters of the phantom.

Wavelength (nm)	*μ*_*a*_ (mm^−1^)	*μ*_*s*_′ (mm^−1^)
671	0.00029	1.08
710	0.00051	1.11

**Table 3:** Eigenvalues of the correlation matrix of the measured data.

Case	Eigenvalues (×10^4^)					
1	30.2451	0.0227	0.0000	0.0000	0.0000	0.0000
2	74.9827	0.0397	0.0000	0.0000	0.0000	0.0000
3	192.6189	0.0337	0.0000	0.0000	0.0000	0.0000
4	70.1423	0.0048	0.0000	0.0000	0.0000	0.0000

**Table 4:** RMSEs of unmixed images for Experiment 1.

Case	PCA	ICA	CAMNS
1	22.8498	24.1539	10.2471
2	24.9656	20.9899	10.7717
3	25.6323	23.7272	11.6476
4	23.4577	18.7858	13.1284

**Table 5:** RMSEs of unmixed images for Experiment 2.

Case	PCA	ICA	CAMNS
1	42.8651	40.2336	8.2746
2	20.1198	14.6347	9.4369
3	18.9276	11.1825	10.9726
